# Comparison of ethanol concentrations in the human brain determined by magnetic resonance spectroscopy and serum ethanol concentrations

**DOI:** 10.1007/s00414-020-02325-w

**Published:** 2020-06-10

**Authors:** Annette Thierauf-Emberger, Judith Echle, Michael Dacko, Thomas Lange

**Affiliations:** 1 Institute of Forensic Medicine, Medical Center - University of Freiburg, Albertstraße 9, 79104 Freiburg, Germany; 2 Medical Physics, Department of Radiology, Medical Center - University of Freiburg, Freiburg, Germany

**Keywords:** Brain, Ethanol, Magnetic resonance spectroscopy, Serum

## Abstract

**Aims:**

Ethanol is a widespread substance that inherits desired effects, but also negative consequences with regard to DUI or battery. Where required, the ethanol concentration is usually determined in peripheral venous blood samples, while the brain is the target organ of the ethanol effects. The aim of this study with three participants was the determination of the ethanol concentration in functionally relevant regions of the brain and the comparison with serum ethanol concentrations.

**Design:**

After the uptake of ethanol in a calculated amount, leading to a serum ethanol concentration of 0.99 g/L, the ethanol concentrations in the brain were directly analyzed by means of magnetic resonance spectroscopy on a 3 Tesla human MRI system and normalized to the water content. The measurement voxels were located in the occipital cortex, the cerebellum, the frontal cortex, and the putamen and successively examined. Intermittently blood samples were taken, and serum was analyzed for ethanol using HS-GC-FID.

**Findings and conclusions:**

Ethanol concentrations in brain regions normalized to the water content were lower than the measured serum ethanol results and rather homogenous within the three participants and the various regions of the brain. The maximum ethanol concentration in the brain (normalized to water content) was 0.68 g/L. It was measured in the frontal cortex, in which the highest results were gained. The maximum serum concentration was 1.19 g/L. The course of the brain ethanol curve seems to be flatter than the one of the serum ethanol concentrations.

## Introduction

In many parts of the world, the consumption of alcoholic beverages is firmly established in the society. According to the WHO, the highest levels of per capita alcohol consumption are observed in countries of the WHO European Region [1]. In contrast to that, the highest rates of lifetime abstainers are reported from the WHO African, Eastern Mediterranean, and South-East Asia regions [1]. In Europe, the recorded alcohol per capita (age 15+) consumption is estimated at 9.8 L for 2016 (3-year averages) [1]. Germany, a country with a permissive alcohol culture, reaches a higher estimated total amount per capita per year (13.4 L) [1].

Ethanol can inherit positive effects like cheerfulness and relaxation, but also negative short- or long-term consequences with regard to DUI (“driving under the influence”/drunk driving) or battery on the one hand side and alcohol use disorders and dependence on the other hand side. In 2018, among 2.6 million road accidents 15,681 occurred under the influence of drugs and alcohol in Germany [2]. As a consequence of road traffic injuries, 1424 deaths are alcohol-attributable, representing age-standardized death rates of 6.0% for males and 2.4% for females respectively in Germany in 2016 [1].

Positive and negative effects of ethanol consumption are located in the brain. The brain is with all its parts the major organ for the acute impacts: Motor center failure symptoms as well as acute psychic effects are results of the presence of ethanol in the brain. The onset of effects takes place in different parts of the brain. An impairment of the coordination is associated with the cerebellum, while the equilibrium is controlled in the motor centers of the brain. Disinhibition is located in the frontal lobe and visual disorders among others in the visual cortex [3].

In daily routine, when legal consequences are pending, in Germany the ethanol concentrations are determined in peripheral venous blood. So far, not much data has been published with regard to the comparability of brain and blood ethanol concentrations in living people. The ethanol concentration can be measured directly in the brain by means of proton magnetic resonance spectroscopy (MRS). Previous studies on brain ethanol concentrations have mainly been performed by radiologic and addiction medical working groups. They focused on the development and optimization of the measurement technique [4–6], on detectable changes due to repeated or chronic ethanol uptake [4, 7, 8], and the analytical comparison of blood and brain ethanol concentrations [5, 9].

Differing results have been published with regard to the comparison of matrices: Mendelson and Kaufman reported higher blood than brain ethanol concentrations [7, 9] while Hetherington observed similar concentrations or higher results in the brain tissue [5]. Differences between the matrices are explained by an invisible ethanol pool with extremely short T2 relaxation, arising from the interaction of ethanol with membrane lipids [7, 10]. The choice of echo time (TE), differences in tissue composition (fractions of gray matter, white matter, and cerebrospinal fluid), and different quantification references may be responsible for varying MRS results in the literature [7, 9, 11]. Normalization to the water content of the analyzed brain tissue was not performed in these studies. Hetherington et al. presented kinetic studies, which made methodical aspects and the comparison between blood and brain a subject of discussion [5]. Measured brain ethanol concentrations in this publication did not relate to brain tissue, but to the aqueous components of the measurement voxel [5].

The aim of this study was the comparison of ethanol concentrations in different and functionally relevant locations of the brain—after normalization to water content—and serum ethanol concentrations. The frontal and occipital cortex of the brain, the putamen, and the cerebellum were chosen as regions of interest for the abovementioned reasons. Repeated measurements were performed for each area.

## Materials and methods

### Experimental set-up

The study was approved by the Ethics Committee of Freiburg University (project nr. 101/17). All study participants gave their written informed consent after complete description of the study to the test persons. The subjects were recruited via notice boards.

Drinking experiments were performed as follows: After at least 2 days of abstinence from alcoholic beverages and 2 h after a light breakfast a void blood sample was collected prior to the start of the experiment. Each volunteer then drank an individually calculated amount of vodka (40 vol%, optionally diluted with lemonade) within 30 min, aiming at a serum ethanol concentration of 0.99 g/l, corresponding to a blood ethanol concentration of 0.8 g/kg. The calculation was based on Widmark’s equation. At the end of the drinking period, another blood sample was taken.

Then, the subjects were positioned in the MR scanner. After the acquisition of an anatomical dataset for voxel positioning, four MRS measurements were successively performed in the occipital cortex, the cerebellum, the frontal cortex, and the putamen. After measurement of the occipital and cerebellar voxels, a venous blood sample was taken while the subject stayed inside the scanner. After measurement of the remaining voxels (frontal cortex and putamen), another blood sample was taken and the subject was given a short break of 15 min and the opportunity to drink some water.

The experiment was continued with another measurement cycle (anatomical scan, measurements of the occipital cortex and cerebellum, blood sampling, measurements of the frontal cortex and putamen, blood sampling). After the second measurement cycle, the breath ethanol concentration was determined using mobile handsets (Draeger Alcotest 6510, Lübeck, Germany). Once the test person showed less than 0.15 mg/L of breath ethanol, they were allowed to leave the experimental setting.

### Chemicals and instrumentation

#### Determination of the blood ethanol concentration

For blood sampling, safety cannulas (21 G) and monovette (S-Monovette® 9 ml, serum with clot activator from Sarstedt (Nümbrecht, Germany)) were used. Serum ethanol concentrations were measured using HS-GC-FID (headspace gas chromatography–flame ionization detection) with t-butanol as internal standard. Ethanol determination was performed using linear calibration with aqueous calibrators containing 0.1, 0.2, 0.5, 1, 2, 3, 4, and 5 g/L of ethanol. The lower limit of quantitation (LLOQ) was the lowest calibrator’s concentration (0.1 g/L for serum or 0.08 g/kg for blood). The method used has been fully validated. For a better comparability of the results, the serum ethanol concentration was preferred over the blood ethanol concentration in this study.

#### Determination of the brain ethanol concentration via MRS

**Fig. 1:**
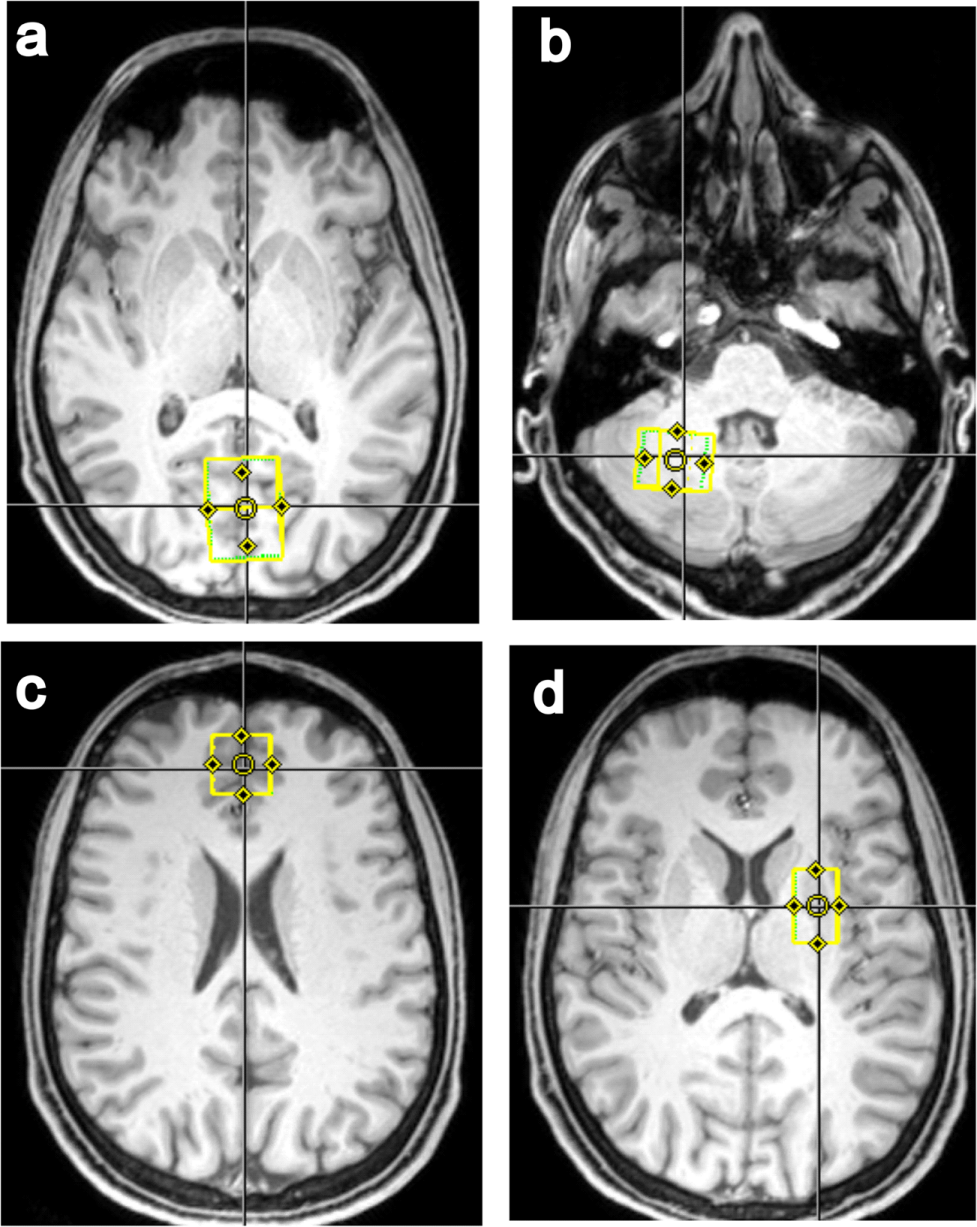
Localization (yellow box) of the voxels: **a** occipital cortex, **b** cerebellum, **c** frontal cortex, and **d** putamen

**Fig. 2:**
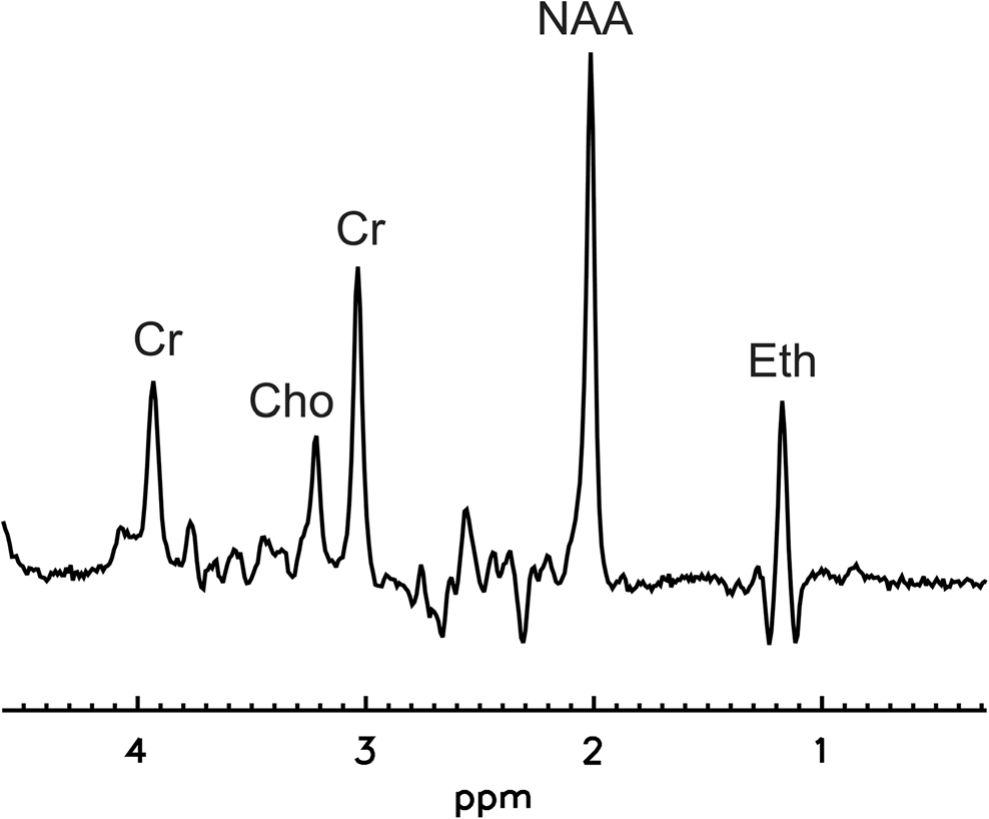
MR spectrum from the occipital cortex showing the ethanol resonance (Eth) as well as the resonances of the brain metabolites N-acetyl aspartate (NAA), creatinine (Cr), and choline-containing compounds (Cho)

The MRS measurements were performed with a 3 T Prisma MR system (Siemens Healthineers, Germany), using a 64-channel receive coil. Initially, an anatomical measurement with a T1-weighted magnetization-prepared rapid gradient echo (MPRAGE) protocol was conducted for positioning of the MRS voxels and tissue segmentation. Spectroscopic data were successively acquired from four voxels located in the occipital cortex (15.6 ml), the cerebellum (8 ml), the frontal cortex (8 ml), and the putamen (7.5 ml) (Fig. 1). The MRS measurements were conducted with a single voxel sLASER protocol [12] of approximately 7 min, using an optimized echo time of 74 ms for ethanol detection. Additionally, after each MRS measurement, a 50-s water-unsuppressed reference scan was performed for absolute quantification via the internal water reference method [13]. The brain ethanol concentrations were determined from the acquired MRS data via a linear combination of metabolite basis spectra (LCModel) [14]. An example of a spectrum is shown in Fig. 2. From the anatomical dataset, gray matter (GM), white matter (WM), and cerebrospinal fluid (CSF) tissue fractions of the measurement voxels were determined via segmentation with the Freesurfer software [15]. These tissue fractions were used for estimating the water content of the MRS measurement voxels, assuming water concentrations of 42.9 mol/L (78 %) for GM, 35.8 mol/L (65 %) for WM, and 53.4 mol/L (97 %) for CSF. Since brain ethanol was assumed to be distributed only within the aqueous components, concentrations were normalized to the overall water volume fraction of the voxel, as previously suggested by Hetherington et al. [5]. For obtaining the ethanol concentrations without water normalization, the values have to be multiplied by the corresponding water content of the voxel. For lack of reliable relaxation values at 3 T, no relaxation correction was performed.

## Results

Three healthy, male subjects (V, volunteer) participated in this study (V1: 26 a, 173 cm, 62 kg, drinking amount: 0.15 L; V2: 29 a, 183 cm, 69 kg, drinking amount: 0.17 L; V3: 24 a, 185 cm, 82.5 kg, drinking amount: 0.20 L). The zero samples were all negative for ethanol. The serum ethanol concentrations in the samples taken at the end of the drinking period (T1) showed great differences: V1 presented the lowest concentration with 0.26 g/L while V2 had a high serum ethanol concentration at the end of the drinking period (1.19 g/L, V3: 0.54 g/L). The subsequent blood sampling (T2) occurred about 40 min after T1; V1 showed a steep, V3 a moderate increase of serum ethanol concentration. The serum ethanol concentrations of V2 were rather steady within the first 70 min (T1–T3). In V1, the maximum concentration was measured at this point of time (T3, 1.14 g/L); V3 presented similar results at T3, T4 (about 130 min after T1), and T5 (about 160 min after T1), suggesting a maximum ethanol concentration between T3 and T4. V1 and V2 showed similar concentrations from T3 on—after differing curve progression after the ethanol consumption. The ethanol concentrations in the three participants were very similar at T5 (about 160 min after T1).

Standard error estimates for the measured metabolite concentrations were provided in the LCModel fits via Cramér-Rao lower bounds (CRLBs) [16]. These are lower bounds for the precision of the quantification results and do not account for systematic errors, e.g., arising from an imperfect fitting model. The various single voxel measurements performed in this study yielded CRLBs between 3 and 12% for the determined ethanol concentrations. The concentrations of brain ethanol showed smaller differences in the three participants. Neither over time nor in the various locations largely differing results were measured: In the first occipital measurement, the concentrations ranged between 0.56 (V3) and 0.62 g/L (V1), in the first cerebellum analysis between 0.50 (V3) and 0.62 g/L (V1). This is the location of the largest variation. The first frontal lobe measurement showed a smaller range (0.61 g/L in V2, 0.62 g/L in V3, and 0.68 g/L in V1). The last analysis of this measuring cycle produced the lowest results in all three participants for the putamen (0.43 g/L in V3, 0.49 g/L in V1 and 0.51 g/L in V2). In the second measuring cycle, the highest results were again observed in the frontal cortex (penultimate measurement, 0.44–0.67 g/L), while the lowest concentrations were detected in the last measurement, in the putamen (0.22-0.47 g/L). The results of the penultimate measurement (frontal lobe) were higher than in the two preceding measurements (occipital cortex, 0.42–0.59 g/L and cerebellum, 0.38–0.42 g/L). The results of the study are shown in Table 1.

**Table 1:** Brain and serum ethanol concentrations in the three participants (normalized to the overall water volume fraction of the voxel)

Localization	Ethanol (g/L)			Point of time (approximately)
	V1	V2	V3	
Serum	0.26	1.19	0.54	End of drinking (T1)
Occipital cortex	0.62	0.60	0.56	
Cerebellum	0.62	0.58	0.50	
Serum	1.03	1.08	0.80	+ 40 min (T2)
Frontal cortex	0.68	0.61	0.62	
Putamen	0.49	0.51	0.43	
Serum	1.14	1.12	0.87	+ 70 min (T3)
Occipital cortex	0.47	0.42	0.59	
Cerebellum	0.39	0.38	0.42	
Serum	0.98	0.98	0.87	+ 130 min (T4)
Frontal cortex	0.51	0.44	0.67	
Putamen	0.22	0.23	0.47	
Serum	0.84	0.84	0.85	+ 160 min (T5)

In this study, the composition of each voxel with regard to WM, GM, and CSF was determined based on segmentation of the MPRAGE dataset. The percentages are shown in Table 2.

**Table 2:** Percentage of gray matter (gm), white matter (wm), and cerebrospinal fluid (csf) as well as the water content of the voxel (wc) in the measuring voxels, assuming water concentrations of 42.9 mol/L for GM, 35.8 mol/L for WM, and 53.4 mol/L for CSF for normalization

Localization	Percentage (%)											
	V1				V2				V3			
	gm	wm	csf	wc	gm	wm	csf	wc	gm	wm	csf	wc
Occipital cortex	46	50	4	72.3	53	42	5	73.5	40	58	3	71.8
Cerebellum	80	20	0	75.4	86	14	0	76.2	78	22	0	75.1
Frontal cortex	11	67	22	73.5	11	67	22	73.5	11	81	8	69.0
Putamen	87	13	0	76.3	91	9	0	76.8	83	17	0	75.8
Occipital cortex	48	48	4	72.5	54	42	4	73.3	41	56	3	71.3
Cerebellum	83	17	0	75.8	89	11	0	76.6	77	23	0	75.0
Frontal cortex	16	64	20	73.5	11	66	23	73.8	12	79	9	69.4
Putamen	85	15	0	76.1	88	12	0	76.4	77	23	0	75.0

## Discussion

This study addressed the direct determination of ethanol in functionally relevant regions of the brain and represents a new approach in the connection of MRS and effects of alcohol. For clinical and forensic purposes, ethanol concentrations are mainly determined in serum and blood while the target organ of the ethanol impact is the brain.

Several postmortem studies focused on the ratio between blood and brain ethanol concentrations (not normalized to the water content) [17–20]. Bonventre et al. [18] reported a good correlation between blood and brain ethanol concentrations and suggested a formula to calculate the brain concentration: C brain (g/100 g) = 0.487 C blood (g/dL) + 0.055. These authors observed a concentration ratio between blood and brain of 0.97 for blood levels of 0.1 g/dL and of 1.32 for blood levels of 0.2 g/dL [18]. The ratio published by Hine is 1.53 [20], but also much higher ratios in the range of 2 to 6 have been observed [18].

As mentioned above, the question of blood and brain ethanol concentrations has also been addressed by means of MRS with differing results. Higher blood than brain ethanol concentrations as well as similar or even lower blood concentrations have been observed in the few existing studies in living humans, primates, and rats [5, 7, 9]. It should be noted that ethanol concentrations measured with MRS strongly depend on the sequence parameters and quantification methods (e.g., the reference metabolite). In our study, the serum ethanol concentrations measured at the time points T2–T5 clearly exceeded the brain ethanol concentrations. Only the blood serum concentrations of V1 and V3 measured at time point T1 were roughly equal to or even smaller than the brain ethanol concentrations measured shortly after. However, it must be noted that this was during the ethanol resorption phase when the delays between blood and brain measurements have a substantial influence.

Concentration differences within the brain have been studied in postmortem examinations (without normalization to the water content) [21–23]. Moore et al. [17] compared tissue from the occipital lobe and the cerebellum with variable ratios (cerebellum/occipital lobe) between 0.4 and 2.1. This range was confirmed by other authors [22, 23]. Differences in the ethanol distribution were attributed to a differing water content of the gray and white matter and a better vascularization and blood flow of the gray matter [17].

The ethanol concentrations of the various anatomical regions in our study were rather similar. It has to be taken into account that in contrast to postmortem examinations our study did not analyze the various sites at a point of time, but rather successively. The differences determined are therefore ascribable also to the time passed between two measurements (approximately 7 min). Due to the limited precision of the brain ethanol quantification results, concentration differences conditional on the localization might at best be apparent for the frontal cortex on the one hand and the putamen on the other hand. In all three test persons, the voxels from the frontal lobe had the by far highest percentage of cerebrospinal fluid. It has to be noted that ethanol resolved in CSF is assumed to have a much larger T2 relaxation constant than ethanol resolved in WM and GM. Since no relaxation correction was performed in this study (for lack of reliable values), the different voxel compositions might thus explain the bias for the frontal cortex. For a better comparison of measured ethanol concentrations, this issue should be addressed with dedicated ethanol T2 measurements in voxels with various tissue compositions, as demonstrated by Sammi et al. with a regression analysis for a field strength of 4 T [6], but also taking into account the CSF signal fraction.

The few data points of the serum ethanol curve are rather different in the three participants, at least in the ascending part of the curve. In contrast to that, the brain ethanol concentrations are more homogenous in the test persons. The course of the brain ethanol curve seems to be flatter than the one of the serum ethanol concentrations. Concentration differences tracing the typical course of the blood ethanol curve with resorption and elimination were weakly defined. With regard to kinetics, further research is necessary in a bigger collective. The results of this study are limited by the small number of participants and the rather low precision of the MRS results.

## Data Availability

Data is fully demonstrated in the article. Row data can be requested from the authors.
